# Viral niche-partitioning: comparative genomics of giant viruses across environmental gradients in a high Arctic freshwater-saltwater lake

**DOI:** 10.1093/ismeco/ycae155

**Published:** 2024-12-08

**Authors:** Thomas M Pitot, Catherine Girard, Josephine Z Rapp, Vincent Somerville, Alexander I Culley, Warwick F Vincent, Sylvain Moineau, Simon Roux

**Affiliations:** Département de Biochimie, de Microbiologie et de Bio-informatique, Université Laval, 2325 rue de l’Université, Québec, QC G1V0A6, Canada; Takuvik International Research Laboratory, Université Laval, 2325 rue de l’Université, Québec, QC G1V0A6, Canada; Centre d’études nordiques (CEN), Université Laval, 2325 rue de l’Université, Québec, QC G1V0A6, Canada; Institut de Biologie Intégrative et des Systèmes, Université Laval, 2325 rue de l’Université, Québec, QC G1V0A6, Canada; Department of Energy - Joint Genome Institute, Lawrence Berkeley National Laboratory, 1 Cyclotron Road, Berkeley, CA 94720, United States; Centre d’études nordiques (CEN), Université Laval, 2325 rue de l’Université, Québec, QC G1V0A6, Canada; Département des Sciences Fondamentales, Université du Québec à Chicoutimi, 555 Boulevard de l'Université, Chicoutimi, QC G7H 2B1, Canada; Centre d’études nordiques (CEN), Université Laval, 2325 rue de l’Université, Québec, QC G1V0A6, Canada; Département de Biologie, Université Laval, 1045 Avenue de la Médecine, Québec, QC G1V0A6, Canada; REV Ocean, Oksenøyveien 10, Lysaker 1327, Norway; Département de Biochimie, de Microbiologie et de Bio-informatique, Université Laval, 2325 rue de l’Université, Québec, QC G1V0A6, Canada; Institut de Biologie Intégrative et des Systèmes, Université Laval, 2325 rue de l’Université, Québec, QC G1V0A6, Canada; Takuvik International Research Laboratory, Université Laval, 2325 rue de l’Université, Québec, QC G1V0A6, Canada; Centre d’études nordiques (CEN), Université Laval, 2325 rue de l’Université, Québec, QC G1V0A6, Canada; Pacific Biosciences Research Center, 1800 East-West Road Honolulu, HI 96822, United States; Takuvik International Research Laboratory, Université Laval, 2325 rue de l’Université, Québec, QC G1V0A6, Canada; Centre d’études nordiques (CEN), Université Laval, 2325 rue de l’Université, Québec, QC G1V0A6, Canada; Institut de Biologie Intégrative et des Systèmes, Université Laval, 2325 rue de l’Université, Québec, QC G1V0A6, Canada; Département de Biologie, Université Laval, 1045 Avenue de la Médecine, Québec, QC G1V0A6, Canada; Département de Biochimie, de Microbiologie et de Bio-informatique, Université Laval, 2325 rue de l’Université, Québec, QC G1V0A6, Canada; Institut de Biologie Intégrative et des Systèmes, Université Laval, 2325 rue de l’Université, Québec, QC G1V0A6, Canada; Department of Energy - Joint Genome Institute, Lawrence Berkeley National Laboratory, 1 Cyclotron Road, Berkeley, CA 94720, United States

**Keywords:** polar virology, viromics, meromictic lake, Nucleocytoviricota, metabolic potential

## Abstract

Giant viruses (GVs; *Nucleocytoviricota*) impact the biology and ecology of a wide range of eukaryotic hosts, with implications for global biogeochemical cycles. Here, we investigated GV niche separation in highly stratified Lake A at the northern coast of Ellesmere Island, Nunavut, Canada. This lake is composed of a layer of ice-covered freshwater that overlies saltwater derived from the ancient Arctic Ocean, and it therefore provides a broad gradient of environmental conditions and ecological habitats, each with a distinct protist community and rich assemblages of associated GVs. The upper layer (mixolimnion) had measurable light and oxygen, and contained diverse GVs linked to photosynthetic protists, indicating adaptation to surface biotic and abiotic conditions. In contrast, the saline lower layer (monimolimnion), lacking oxygen and light, hosted GVs associated with predicted heterotrophic protists, some of which are known for a predatory lifestyle, and with several viral genes suggesting adaptation to deep-water anaerobic conditions. Our observations underscore the coupling between physical and chemical gradients, microeukaryotes and their associated GVs in Lake A, and provide insight into the potential for GVs to directly and indirectly impact host metabolism. There were similarities between the genetic composition of GVs and the metabolic processes of their potential hosts, implying co-evolution and niche-adaptation within the lake habitats. Notably, we found a greater presence of viral rhodopsins in deeper water layers, suggesting an evolutionary relationship with potential hosts capable of supplementing their energetic needs to thrive in low energy, anoxic conditions.

## Introduction

Giant viruses (GVs; *Nucleocytoviricota*) have been discovered in a wide range of biomes and ecosystems, and their enormous genetic diversity in the ocean potentially surpasses that of Bacteria and Archaea [1]. Since their discovery and formal description in 2003, there has been growing recognition of their potential ecological importance and role as top-down regulators of eukaryotic host communities, drivers of evolution, and contributors to local and large scale biogeochemical cycles [2–4]. Until recently, our understanding of GV genomes (reaching sizes of several megabases) largely relied on species-level isolates, which were primarily obtained through laboratory-based co-cultivation with a limited number of cultured protists and algae. While these isolates demonstrated that GVs can possess thousands of genes, many of which appear to have been acquired from diverse cellular and viral lineages [5, 6] and involved in various metabolic processes, they did not capture the full extent of GVs diversity and their ecological contexts.

Recent analyses of large numbers of GV metagenome-assembled genomes (GVMAGs) have significantly expanded our understanding of this diversity. These GVMAGs confirmed that GV genomes are remarkably varied and encode thousands of genes and that many may play roles in various microbial and metabolic processes, including nitrogen cycling, photosynthesis, and nutrient assimilation [7, 8]. Importantly, genes involved in glycolysis and the tricarboxylic acid (TCA) cycle have been identified in GV genomes, suggesting that these biological entities can influence crucial aspects of their host’s metabolic processes [9].

One of the key ecosystem functions of microbes in the aquatic environment is the harvesting of light via rhodopsin. Recent research has indicated that viral rhodopsins (VirRs), also found in GVs, are prevalent in marine metagenomes [10]. VirRs have been found to be cation channels (i.e. channelrhodopsins) [11, 12]. Viruses might utilize channelrhodopsins to influence the phototaxis and swimming behavior of their hosts, potentially guiding infected cells toward nutrient conditions that are more conducive to viral production [13, 14]. VirRs are present in metagenomes from the ocean surface down to the deep chlorophyll maximum (DCM), suggesting that these light-dependent energy transfer systems are common features in GVs associated with photosynthetic and phagotrophic unicellular eukaryotes [10].

While GVs have been detected in all aquatic environments [15–17], recent metagenomic surveys revealed their remarkable diversity and richness in polar lake ecosystems as well as strong patterns of endemism [18]. Polar lakes have short, truncated food webs [19], and the absence of grazers or higher trophic organisms may give GVs a disproportionate role in these systems. While diverse, little is known about how GV genotypes are partitioned across environmental gradients. Meromictic lakes provide ideal model environments to address this question because of their heterogeneous vertical stratification. These vertical gradients are especially pronounced in meromictic lakes in the polar regions, where their bottom waters are sometimes derived from seawater trapped by isostatic uplift, and their surface is composed of low conductivity, freshwater derived from melting of the adjacent cryosphere (snow, glaciers). The perennial ice cover of these lakes in combination with their strong density gradients prevents the mixing of the water column and allows strong physical and chemical gradients to persist, in turn giving rise to striking changes in microbial community structure with depth [19].

Here, we analyzed the community composition, abundance, and metabolic potential of GVs in Lake A (83°00′N, 75°30′W), a 3000 year-old, marine-derived meromictic lake on the far northern coast of Ellesmere Island in the Canadian High Arctic (details in [20]). We hypothesized that *Nucleocytoviricota* populations would show niche partitioning across the physicochemical gradients of Lake A, with changes in abundance, genetic composition, and diversity that closely correlate with depth-dependent changes in the local eukaryotic populations. Specifically, we anticipated finding viruses infecting microalgae and other photosynthetic microeukaryotes in the upper water column while GVMAGs in deeper layers may be associated with sinking particles as well as GV populations adapted to deep-water hosts and conditions, similar to the recently described dark-water GV community [21]. By analyzing the taxonomic and functional diversity of GV populations across these gradients, we aimed to identify the origin, potential hosts, and possible impact on microbial metabolism of the different GV populations of Lake A.

## Materials and methods

### Data acquisition

**Figure 1:**
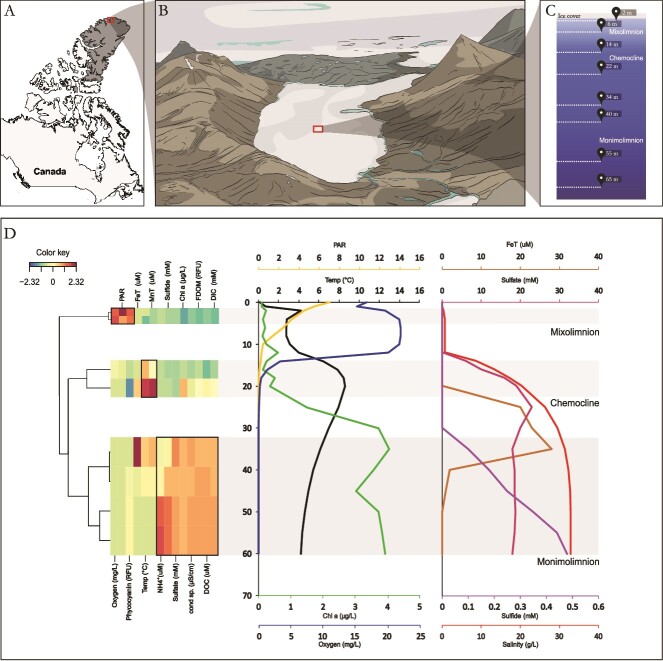
Lake A limnology. (A) Location of meromictic Lake A, in a non-glacierized catchment on the northern coast of Ellesmere Island, Nunavut, Canada. (B) Illustration of the Lake A watershed and the location of water column sampling. (C) Biological sampling depths in the water column (eight depths). (D) Heatmap and clustering dendrogram of physicochemical parameters with water depth. A continuous profile of measured and interpolated data values from measurements at adjacent depths is also presented across the identified layers of the lake. PAR, in mol photons m^−2^ day^−1^.

Lake A, located on the northern coast of Ellesmere Island (Fig. 1A and B), was sampled on 18 July 2017. Its water column extends to a depth of 128 m and exhibits a pronounced stratification. The uppermost freshwater layer (from surface to 14 m depth), defined as the mixolimnion, is characterized by low conductivity, measurable irradiance levels and oxygenation. This layer primarily originates from snowmelt-fed inflowing streams. Beneath the mixolimnion is a transition zone with steep salinity and other chemical gradients (the chemocline), and finally, the deepest and bottom layer (from 22 m), the monimolimnion, is derived from the ancient Arctic Ocean and is marked by high salinity and the absence of oxygen and measurable light. The lake contains microbial communities that are strongly partitioned by these environmental gradients [22–25]. Water samples were collected in triplicate from eight sampling depths, where sharp changes in the geochemical composition of the water column were observed (at 2, 6, 14, 22, 34, 40, 55, and 65 m). Triplicates were taken from three independent 24-cm-diameter holes drilled through the 0.6 m summer ice cover near the middle of the lake [22]. Dissolved oxygen, salinity, chlorophyll *a*, and temperature spanning the entire water column were measured using a YSI-EXO2 profiler (Xylem, Inc. Yellow Springs, USA), which measures all red fluorescing particles excited by blue light, without discriminating between pigments. Photosynthetically active radiation (PAR), SO_4_^2−^, H_2_S, total Mn, and total Fe were obtained from previous studies [22, 23, 26]. Water samples were collected using a 7 L Limnos water sampler (Limnos.pl), and underwent a two-step filtration process in the field laboratory. Samples were first filtered through SterivexTM filters with a pore size of 0.22 μm (cellular and GV size fraction). Sterivex filtrates were subsequently filtered through AnotopTM filters with a pore size of 0.02 μm (traditional viral size fraction). The filters were stored in the field at −50°C and at −80°C following field work, until nucleic acid extraction. Deoxyribonucleic acid (DNA) extractions were performed as described elsewhere for Sterivex [27] and for Anotop filters [28]. Four metagenomes were constructed per sampled depth using one Sterivex filter and all triplicate Anotop filters. Metagenomic library preparation and Illumina sequencing were performed as described previously [22].

### Characterization of ecological habitats

To assess potential ecological habitats based on physicochemical gradients in the lake, we used Clustered Image Maps (CIM) with the (s) principal component analysis single omics method to visualize as a heatmap the clustering of physicochemical parameters along depth (mixOmics v.6.24.0) in R v.4.3.1 [29]. Distances were calculated with default dist.method = “euclidean”. Clustering of metadata as primary factors of corresponding depth was processed with default clust.method = “complete”. Clusters of environmental variables on the dendrogram were considered as potential habitats.

### Giant virus and eukaryote diversity

GVMAGs were previously assembled and refined in [18]. Briefly, GVMAGs were assembled by binning with a minimum length of 75 000 base pairs from contigs (≥ 1500 base pairs) using metabat2 (v.2.15) [30]. The generated bins underwent dereplication [31] and processing through the identification and taxonomic classification tool GVClass (v.0.9.3) [32]. Briefly, highly conserved proteins from orthologous groups were detected and used to build consensus single-protein phylogenies to ensure strong taxonomic classification. GVMAGs were identified and assigned to a phylum with GVClass according to a conservative approach based on the consensus of single protein trees built from the nine GV orthologous groups (GVOGs). Here, only GVMAGs with a minimum of three GVOGs (“gte3” in GVClass) were selected for downstream analyses, resulting in a dataset of 96 GVMAGs. For further quality control, GVMAGs were screened for the presence of Uni56 unique (universal cellular housekeeping genes), presence of GVOG9 marker genes, and duplication factor of GVOG7, all obtained from the GVClass output. Cutoffs were respectively, Uni56 < 10, GVOG9 ≥ 4, GVOG7df ≤ 3. To further assess taxonomic classification, all GVMAGs were run through TIGTOG (Taxonomic Information of GVs using Trademark Orthologous Groups), a machine learning-based approach to predict GVs taxonomy [33]. Finally, a last round of quality control was run on GVMAGs with ViralRecall to filter potential cellular contamination [34]. Read depth coverage was calculated as read depth coverage = sum (contig length ^*^ contig average total depth)/GVMAG total length. To account for reads mapping to shared genes across different GVMAGs, a GVMAG was considered to be “present” in a sample if at least 70% of its bases were covered in this sample, and “absent” otherwise, with its coverage in this sample thereafter considered to be 0. We applied this stringent coverage threshold of 70% to avoid overestimating the distribution of GVMAGs, prioritizing the reduction of false positives (FP) over false negatives (FN). This approach minimizes spurious mapping data and ensures a conservative estimate of GVMAG presence across samples. Normalized and filtered (based on % of bases covered) GVMAG coverage values were then used to build a GVMAGs sample-wide abundance table. For further quality control, GVMAGs were screened for the absence of a defined set of 56 universal cellular housekeeping genes and the presence of a set of nine GVOGs as shown previously [18].

To assess whether large filters with a pore size of 0.22 μm effectively filtered out all GVs, we applied a ratio of 1 to −1 to compare the recovery of individual viruses across the two size fractions from the abundance table. A ratio of 1 or − 1 indicated that the GVMAG was exclusively observed in either the cellular or viral size fraction, respectively. Ratios ranging from 0.99 to −0.99 were associated with a higher abundance in either the large or small size fraction. The ratio was calculated as follows: sum (GVMAG RPKM per Anotop [small filter]) - sum(GVMAG RPKM per Sterivex [large filter])/100.

To show similarities and differences between samples, the abundance table was transformed into a binary matrix to determine the presence or absence of GVMAGs in samples and an UpSet plot was produced with the R package (UpSetR; v.1.4.0) [35]. The relative abundances of every GVMAGs was also plotted with geom_bar() in (ggplot2; v.3.4.2) [36] to show variations in population composition with depth.

Finally, we calculated the z-score (z = sample abundance - mean abundance^*^standard deviation) of all GVMAGs across every sample. The highest z-score value was used as a proxy to determine what was the most probable habitat and subsequent ecological niche of GVMAGs.

As GVs infect eukaryotic hosts, we investigated the eukaryotic populations across all eight depth samples within the water column of Lake A. To extract the 18S ribosomal RNA (18S rRNA) from the cellular size fraction sequencing raw reads, we employed the methods described in Pitot et al. [18]. Specifically, we used BBMap v.38.93 [37] and the SILVA SSU Ref NR 99 database 138 as references for the extraction of 18S rRNA from the reads [38]. This extraction was performed using phyloFlash v.3.4 with default settings, including the parameter “–almosteverything” and a clustering threshold of 98% identity. The last-common-ancestor consensus of the top hits was utilized to estimate taxonomic affiliations, and read counts were used to generate an overview of micro-eukaryote community composition across the samples. To ensure consistency in reported taxonomic ranks, we conducted manual curation of the classifications.

Relative abundance-correlation between GVMAGs and eukaryotes in the water column was measured with the R package (mixOmics; v.6.24.0) [29] using the sparse projection to latent space (sPLS) method and visualized with a correlation circle plot (plotVar()) with a Pearson correlation coefficient cutoff of 0.5 between the relative abundance tables of both GVMAGs and eukaryotes per sample.

### Genomic comparison of giant viruses across habitats

Gene calling was performed with GeneMarkS (v.4.28) [39] using the virus sequence option on all 96 GVMAGs. Functional annotation was done with the eggNOG mapper (v.2.1.12) to assign their functional categories and KEGG pathways against the eggNOG 5 database [40].

Every predicted GV protein was given the normalized coverage of the GVMAG contig they were identified on as abundance. We generated a CIM heatmap illustrating Pearson correlation coefficients between the metadata table and the KEGG pathway relative abundance table. This heatmap was used to visualize fluctuations in the relative abundance of diverse metabolic pathways and highlighted subsets of features within each table that exhibited correlations, indicating potential interactions across sample depth and inferred ecological habitats. A complementary comparative genomic analysis was conducted to measure variation in KEGG pathways abundance between GVMAGs predicted to be adapted to specific habitats and was illustrated with a ggtern plot from ggtern (v.3.3.2) R package [41].

A restricted analysis was also performed with only contigs predicted as viral instead of the entire GVMAGs to verify that the signal observed was not due to a contamination of GVMAG by sequences of cellular origin. The prediction and detection of viral contigs was realized by running the full geNomad pipeline (end-to-end command) [42]. GeNomad, as a default setting, employs several post-classification filters to eliminate potential FP. Notably, sequences must possess a virus score of at least 0.7. Options “–cleanup” and “–splits 8” were used for memory and storage purposes.

We examined viral genes for signatures of horizontal gene transfer (HGT) to identify potential host organisms or particular host clades by evaluating the maximum taxonomic affiliation level of every predicted gene embedded in all GV contigs from the eggNOG mapper annotation. We computed the cumulative percentages of all identified taxon and visualized their relative contribution in a treemap chart with treemap() from the R package ggplot2 (v.3.4.2) [43].

The “Nucleocytoviricota Metabolic Profiler” (NuMP; https://github.com/BenMinch/NuMP) program was used to analyze the metabolic and functional potential of GVMAG at every sample depth using Hmmscan, pfam-A database [44] and a set of genes and pathways detected in previous GV genomes as targets [45]. Sample-wise normalized counts per GVMAGs were used for plotting the abundance of targeted genes across all sampled depths, and visualized with a bubble plot using geom_point() in ggplot2 [36].

Finally, we screened our dataset for VirRs using the NuMP output and aligned them with VirRs [10] using goalign with “clean site -i” and “-c 0.5” options [46]. A maximum likelihood phylogenetic tree was constructed using IQ-tree with the options “-alrl 1000”, “-B 1000”, and “-m MFP” to use ModelFinder and automatically determine the best-fit substitution model for our data. The resulting tree was visualized and annotated with iTOL (v.6) [47].

## Results

### Three discrete habitats down the water column

To characterize the different habitats of Lake A, we measured the changes in physical and chemical properties at various depths. These limnological profiles confirmed the well-defined stratification and pronounced gradients in the water column beneath the ice (Fig. 1D). Chemical species were strongly stratified in the water column. First, concentrations of sulfate and total Fe were below detection in the mixolimnion and increased at the chemocline to reach their highest values at, respectively, 25 and 35 m depth in the monimolimnion. The monimolimnion was also characterized by the increase of interpolated sulfite concentrations from 35 to 60 m. Chlorophyll *a* concentrations (μg/L, as the total pool of red fluorescing particles) were highest in the monimolimnion after a first peak at the chemocline (14 m). In a previous study, Antoniades *et al.*, [48] showed that the chlorophyll profile of Lake A is characterized by two types of chlorophyll pigments: algal Mg-tetrapyrrole chlorophyll *a*, dominates from the surface to the bottom of the chemocline, whereas the deep layers of the lake are concentrated in bacteriochlorophyll *a* used by anoxygenic photosynthetic bacteria (Fig. 1D). Considering all chemical profiles, we were able to categorize the eight biological samples into three clusters corresponding to different physico-chemical areas, which we will refer to as “Mixolimnion” (2–6 m), “Chemocline” (14–22 m), and “Monimolimnion” (32–65 m) habitats (Fig. 1D).

### Lake A *Nucleocytoviricota*

The viral metagenomes of Lake A contained many different biological entities. Here, we focused on GVs and specifically on *Nucleocytoviricota*. According to GVClass, the predominant order of GVs in the lake was that of the *Imitervirales*, representing 92.7% (89 out of the 96 GVMAGs) of the total GV community. Most of these were classified as *Mesomimiviridae*, a recently demarcated monophyletic viral family including aquatic algal viruses, such as *Tethysvirus hollandense*, *T. ontarioense*, and *T. raunefjordenense*, infecting haptophytes [49]. Three other orders were observed in the water column, with *Pimascovirales* and *Asfuvirales* being the second and third most prevalent, accounting for relative abundances of 3.1% (3 GVMAGs) and 2.7% (2 GVMAGs), respectively. Additionally, only one GVMAG (1.0%) was associated with the *Pandoravirales* order, while another remained unclassified within the *Nucleocytoviricota* phylum (Supplementary Fig. 1A). Taxonomic classification was further confirmed with TIGTOG with 100% similarity at the phylum level, 98% similarity at the order level, and 82% similarity at the family level (Extended Data Table 1).

Then, we explored the genomic characteristics of the local *Nucleocytoviricota*. The average length of Lake A GVMAGs was 497 kb (SD = 289 kb), with an average GC content of 35.2% (SD = 9.9). Each GVMAG exhibited an average of 484 predicted genes (minimum = 109, maximum = 1876) and demonstrated a high average percentage of coding DNA at 92.5% (SD = 1.7; Supplementary Fig. 1A).

To further confirm the viral origin of the GVMAG sequences of Lake A, we compared these to 56 known cellular markers (UNI56) and 9 GV markers (GVOG9). Lake A GVMAGs displayed a mean of only 2.15 (± 0.8) UNI56 per genome and possessed a mean of 7.7 (± 1.4) GVOG9. When assessed using geNomad, the average percent of contigs flagged as viral was 49.8%. Flagged GVMAGs received an average viral score of 0.93 (± 0.05) for contigs ranging in size from 1502 to 315 030 base pairs (Supplementary Fig. 1C). Finally, the GVMAGs showed a mean duplication factor of GVOG7 (a subset of GVOG9) of 2.15 (± 0.8; Supplementary Fig. 1B). Taken altogether, these values indicate minimal cellular contamination and duplication in the GVMAGs, with the vast majority of genes being encoded by GVs. This result aligns with the ViralRecall analysis, where all GVMAG contigs were screened and achieved a high median score of 8.3 (compared to the cutoff value of 0 recommended by the ViralRecall authors when discriminating between GV and non-GV sequences), further confirming their classification as *Nucleocytoviricota* contigs.

Comparing the distribution of GVMAGs across different filters and subsequent size fractions, all 96 GVMAGs were found in the cellular size fraction, with 22 of them exclusively observed in Sterivex 0.22 μm samples. None of the GVMAGs were unique to the 0.02 μm size fraction (Supplementary Fig. 1D).

GVs typically display a broad coding potential including hundreds to thousands of genes responsible for diverse functions. The presence of genes associated with cellular life (both eukaryote and prokaryote) in their genomes, underscores the close interactions and numerous HGT events that have occurred through the evolutionary history of GVs [50–52]. We examined the relative taxonomic composition of GVMAGs genes in the lake, and a substantial proportion (60.1%) of these genes had a best hit homologue identified within the Eukaryota domain. Notably, among the genes annotated with eggNOG mapper (61.7% of total genes), many affiliated with Unassigned *Eukaryota*, *Streptophyta*, Fungi, and *Metazoa* were particularly abundant, collectively representing approximately 50% of the GVMAG gene pool. *Chlorophyta*, *Ciliophora*, and *Amoebozoa* in particular emerged as significant contributors to the overall eukaryotic signature (collectively ±6%). Prokaryotic-affiliated homologs comprised only 31.7% of genes, whereas those with a viral taxonomic affiliation represented a minor fraction, accounting for 8.2% of the overall taxonomic composition. The other fractions were genes with taxonomic affiliation to taxa observed <50 times in the overall pool of taxa (Supplementary Fig. 1E).

### Depth distributions and correlations between giant viruses and eukaryotes

**Figure 2:**
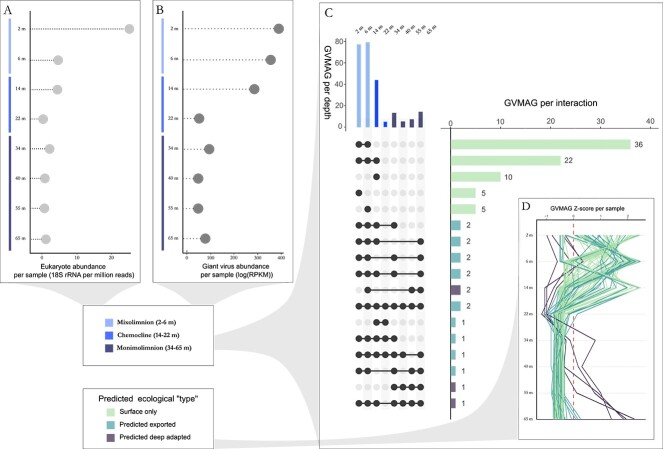
Distribution of GVMAGs and eukaryotic cells in the water column. (A) Distribution of the absolute abundance of eukaryotic cells in the water column. (B) Distribution of the absolute abundance of GVMAGs in the water column. (C) Upset plot of the distribution of GVMAGs along the Lake A water column. (D) Z-score of every GVMAG (96 in total) in all depth samples used as a proxy to determine the most probable habitat and subsequent ecological niche they inhabit.

We explored the diversity and distribution of eukaryotic taxa (estimated from 18S rRNA sequences) and GVMAGs across all 8 samples and 3 layers (mixolimnion, chemocline, and monimolimnion) throughout the water column. GV and eukaryote distribution was closely aligned, characterized by a decline in abundance and diversity from the surface to the monimolimnion (Fig. 2A and B). The highest abundance and greatest diversity were observed in the mixolimnion) for both groups, followed by an initial decrease at 6 and 14 meters. A second significant decline occurred between 14 and 22 meters, after which their abundance remained consistently low and stable in the monimolimnion (Fig. 2A and B). The majority (46) of the 96 GVMAGs were exclusively identified in the upper ecological habitat at 2 and 6 meters (“surface only”), while 11 others were specifically observed in the chemocline, with the majority (10) exclusively detected at 14 m. Ultimately, only one GV was detected exclusively in the deepest ecological habitat. The rest of the GVs exhibited a more generalist distribution, spanning across two or more habitats (Figs 2A and3). Among the GVMAGs detected in the monimolimnion, we observed two distribution patterns (Fig. 2D): a small number of GVMAGs displayed maximal abundance in the mixolimnion and decreased with depth, which may indicate an association of these GVMAGs to sinking particles. A second group of GVMAGs were most abundant in the monimolimnion, and were rare or absent in the rest of the water column. Hereafter, we refer to these two distribution patterns as “predicted exported” and potentially “deep adapted”, respectively.

**Figure 3:**
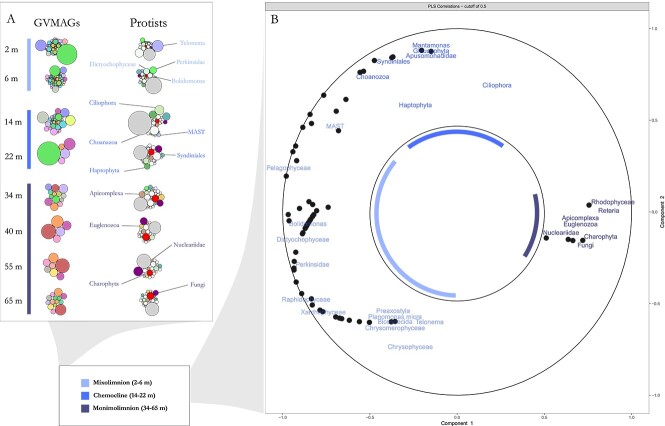
GV and protist co-distribution between habitats. (A) Distribution and relative abundance-correlation of GVMAGs and eukaryotic cells between samples and habitats (mixolimnion, chemocline, monimolimnion) in the water column. The detailed spatial dynamics of eukaryotic cells in the lake is available as Supplementary Fig. 3. (B) Correlation circle plot of sPLS between relative abundance tables of GVMAGs and eukaryotes per sample. A correlation cutoff of 0.5 Pearson correlation coefficient was applied for visibility. Colors in the circle plot refer to habitat where the eukaryotes were most dominant.

Similar to the distribution of GVs, eukaryotes formed three distinct clusters that corresponded to mixolimnion (2–6 m), chemocline (14–22 m), and monimolimnion (32–65 m; Fig. 2C and3A). Only thirteen out of a total of 64 eukaryotic groups were observed across all three habitats and all samples. Among these, some displayed consistent abundance throughout the water column, such as *Choanozoa* which comprised on average 37.9% (± 8.2) of the eukaryotic population at all depths. Other taxa exhibited substantial variation in their relative abundance, such as *Ciliophora*, *Chlorophyta*, and *Haptophyta*, which were most abundant at the chemocline, or *Chrysophyceae*, whose abundance was lowest in the transition zone. Fungi, *Apicomplexa* and *Euglenozoa* were three abundant members to the overall Lake A eukaryotic community, representing 6.4% (± 5.1), 2.5% (± 1.7), and 2.1% (± 1.7), respectively, and all exhibiting greater relative abundances in the deep habitat (Fig. 3).

Correlation analysis of the relative abundance patterns of GVMAGs and eukaryotic cells supported a correlation of distribution patterns of viruses and eukaryotes associated with either mixolimnion, chemocline or monimolimnion (Fig. 3B).

### Metabolic potential and contrasts in the genetic make-up of giant viruses

**Figure 4:**
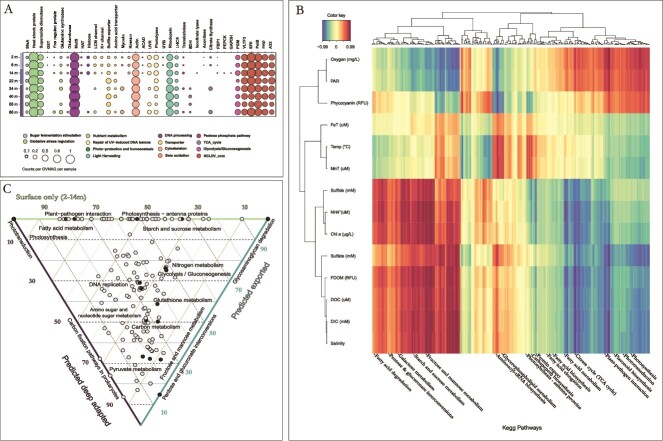
Comparative GVMAGs genomics across ecological niches. (A) Bubble plot of normalized counts of Pfam domains per GVMAG per sample. The bigger the bubble, the higher the proportion of the genes in GVMAGs in the sample. (B) CIM heatmap illustrating Pearson correlation coefficients between metadata and the relative abundance of KEGG pathways across the water column (NH4^+^: ammonium-N). This heatmap highlights subsets of features within each table that exhibit correlations. (C) Ternary plot of the distribution of KEGG pathways between GVMAGs predicted to be adapted to specific habitats/conditions (GVMAGs observed only at the surface, GVMAGs predicted to be exported, and GVMAGs predicted to be adapted to deep conditions).

Among the 37 established genes [45] screened to examine the metabolic potential of GVMAGs, some showed distinct distribution signatures across depths. For example, genes coding for K+ channel transporters were abundant in the monimolimnion but were not detected in samples deeper than 14 m, while photolyase-encoding genes were more prevalent in the mixolimnion, gradually decreasing with depth. Conversely, sulfite exporters increased with depth, particularly in GVMAGs from 22 and 40 m. Additionally, a slight increase in GVMAG genes coding for the SfsA sugar fermentation stimulation protein was observed with depth (Fig. 4A).

To further investigate variations in GVMAG functional potential, we clustered their genetic content throughout the water column based on a comprehensive functional annotation using the KEGG database. Specific groups of metabolic pathways were correlated to each of the three ecological habitats (Fig. 4B). A cluster of KEGG pathways was found almost exclusively in the mixolimnion and contained deduced proteins involved in photosynthesis, plant-pathogen interactions, circadian entrainment, flavonoid biosynthesis, and mismatch repair. Other genes encoding proteins involved in the TCA cycle, glycolysis, fatty acid metabolism, and biosynthesis/elongation were also correlated with the surface habitat (Fig. 4B).

On the other hand, the monimolimnion was characterized by proteins involved in carbon fixation, fatty acid degradation, and mono/disaccharides (fructose, maltose, sucrose, galactose) metabolism and gluconeogenesis.

Unlike the other two habitats, the chemocline did not display a specific signature and seemed to represent more of a combination of flanking habitats with genes coding for proteins involved in photosynthesis-antenna pathway, fatty acid metabolism, biosynthesis and elongation, and nitrogen metabolism (Fig. 4A). Results were near-identical when restricting the analysis to contigs predicted as viral by geNomad instead of the entire GVMAGs (Supplementary Fig. 2).

Finally, we conducted a comparative analysis of the metabolic profiles of the three predicted types of GVMAGs (see above) and observed that pathways associated with photosynthesis and aerobic metabolism were largely constrained to the “surface only” GVMAGs. Meanwhile, comparing metabolic profiles of GVMAGs found in the monimolimnion and considered as “predicted exported” or “deep adapted”, a notable dissimilarity emerged between these two types of GVMAGs. Specifically, pathways related to heterotrophic and potentially anaerobic metabolisms such as pyruvate metabolism, pentose-glucuronate interconversion, and fructose and maltose metabolism showed stronger correlations with the 'deep adapted' GVMAGs (Fig. 4C). Meanwhile, GVMAGs identified as potentially exported from the mixolimnion were enriched in pathways such as nitrogen metabolism as well as starch and sucrose metabolism.

### Distribution of viral-rhodopsin proteins

**Figure 5:**
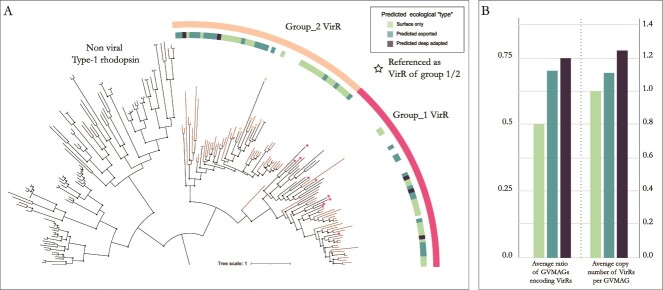
Type-1 rhodopsin phylogenetic tree. (A) Maximum likelihood phylogeny of viral rhodopsin proteins from Lake A GVMAGs, aligned with the type-1 rhodopsin from [10] and distribution across the predicted ecological type. (B) Average distribution of VirRs among GVMAG populations per ecological type.

Given the presence of viral-rhodopsin proteins (VirRs) homologs in our GVMAG dataset, we further investigated the phylogenetic diversity and distribution of these genes down the water column. When included in a phylogeny with known bacterial and VirRs, the VirR homologs from Lake A GVMAGs branched with known VirRs, and away from microbial ones (Fig. 5). The majority (59.5%) of Lake A VirRs clustered with group 2 VirRs, but formed distinct clades from reference genes. When comparing VirRs found throughout the Lake A water column, no clear clustering could be observed, i.e. VirRs sequences from a given habitat were not phylogenetically closer to each other or forming distinct clades.

Independently of their phylogenetic relatedness, the rate of GVMAGs encoding VirRs genes varied between habitats (74.2% vs 50.7% for the GVMAGs observed in monimolimnion and mixolimnion, respectively), as did the number of gene copies, with many GVMAGs in the monimolimnion encoding multiple copies of VirRs (Fig. 5B). Thus, while similar VirRs genes could be detected across the different habitats, “deep-adapted” GVMAGs seemed to encode more and more diverse VirRs than their surface counterparts.

## Discussion

Our observations of the physicochemical parameters in Lake A highlight its pronounced stratification. The transition layer of the chemocline divides the lake into two distinct environments: the oxygenated and irradiated mixolimnion at the surface and the deep and cold monimolimnion. Each of these layers exhibits specific physical and environmental conditions as well as distinct microbial communities [22–25] supporting our hypothesis that the Lake A water column provides a diverse range of habitats, fostering the establishment of unique GV ecotypes within each layer.

Both eukaryotes and GVMAGs exhibited pronounced changes in abundance and diversity with water depth. Despite an overall decrease in both abundance and diversity observed from the water surface to deeper layers, each habitat harbored a distinct GV and eukaryotic community, pointing to habitat-specific host-virus relationships in each layer.

In the mixolimnion, GVMAGs showed strong correlations with autotrophic and photosynthetic heterokont protists (i.e. *Bolidomonas*, *Dictyochophyceae*, *Raphidophyceae*, *Xanthophyceae*) while GVMAGs observed in the monimolimnion were consistently correlated with heterotrophic or mixotrophic protists from the Fungi, *Nucleariidae*, Euglenozoa, and Rhodophyta [23, 53]. *Bangiale*s, for example, are an order of Rhodophyta with a simple morphology, and have been observed in the Arctic. They have plastids allowing for photosynthesis as well as starch and fatty acid biosynthesis, and have various sulfur and nitrogen metabolic pathways [54]. In the chemocline, we observed the presence of both heterotrophic and autotrophic protists alongside GVMAGs. Although their overall relative abundance was low, two families of gliding heterotrophic flagellates, namely the *Apusomonadidae* and the *Mantamonadidae*, exhibited a strong correlation with local GVMAGs.

While many protists were predominantly associated with specific layers within the water column, a significant number from the mixolimnion were also found in the monimolimnion community, albeit in much lower abundance. This may directly result from the timing of our sampling, which occurred following the spring bloom in this Arctic lake and the samples from lower depths likely included cells that had sedimented from this earlier phytoplankton maximum [55].

Still, the distribution of GVMAGs alongside eukaryotic clades supported our initial hypothesis that GV populations vary significantly in terms of their diversity in response to physicochemical gradients in the water column and that these changes correlate to the potential metabolism and ecology of their host. However, we noted that some abundant eukaryotic community members (i.e. *Ciliophora*, *Haptophyta*) were not correlated to any GVMAGs, although known or suspected as potential hosts for GVs in recent studies, including in the Arctic [8, 18]. This suggests that our analysis is possibly limited by an uneven distribution of certain taxonomic groups and a low resolution of eukaryote diversity.

The analysis of gene content and functional annotation revealed substantial variations in the metabolic potential of GVMAGs across the water column. Notably, photolyase-encoding genes were more prominent in GVMAGs from the mixolimnion. Photolyases are enzymes that repair DNA damage caused by exposure to ultraviolet light. A higher frequency of photolyases in the photic zone might be an adaptation to UV stress and could benefit both GV and host fitness. Similarly, K+ channel-related genes were only observed in the mixolimnion and in the 14 m sample of the chemocline. K+ channel is a major osmolyte in the contractile vacuole in freshwater algae to uptake excessive water from the cytoplasm and expel it to the environment to achieve cellular homeostasis [56]. The mixolimnion of Lake A is composed of low conductivity freshwater; salinity starts to increase from 14 m before reaching marine salinity (35 grams per kilogram [g/kg] of seawater, or 35 ppt) in the monimolimnion, consistent with the geomorphological history of the lake [20]. The observation of genes coding for a K+ channel in GVMAGs correlated with the mixolimnion is consistent with the above description suggesting the infection of photosynthetic algae in the freshwater layer.

Similarly, the proportion of sulfite exporters in the GVMAGs corresponded to sulfate distribution in the water column, with a minimum in the mixolimnion and a maximum plateau in the monimolimnion. Although genes for organic sulfur molecule degradation and respiration were found to be abundant and widely distributed in the lake’s phytoplankton community [22], the extrusion of metabolites by infected phytoplankton could potentially have a significant impact on the Lake A sulfur cycle [22].

Therefore, comprehensive functional annotation of GVMAGs indicates that there may be partial adaptation of the GVs to the niches they occupy as evidenced by the differentiation in metabolic proteins along the depth gradient.

GVs infect various eukaryotic hosts, often reproducing in specialized structures known as viral factories in the host cytoplasm, or alternatively within the nucleus where they replicate prior to virion assembly in the viral factories as reviewed in Schulz et al. [8]. Many of these viruses have been shown to encode a variety of metabolic genes that likely contribute to shifts in host physiology and to augment and/or modulate the metabolic capabilities of the infected host cell [9, 16]. Horizontal gene transfer was suggested as one of the main mechanisms for the acquisition of these genes. Recent studies have shown that their encoded complex metabolic makeup have evolutionary histories independent of cellular life, strongly implicating them as important drivers of global biogeochemical cycles [9]. We screened GVMAGs for such potential metabolic genes with the objective to identify their host metabolism and growing capabilities to increase its sustainability and fitness in the ecological niche they occupy.

Genes associated with autotrophic pathways or interactions with phototrophic organisms were notably more abundant in GVMAGs correlated with the mixolimnion (e.g., photosynthesis, TCA cycle, plant-pathogen interaction, fatty acid metabolism). However, we observed a larger number of GVMAGs in the mixolimnion that were also found in the monimolimnion, categorized as “predicted exported” GVs, lacking genes involved in autotrophic metabolism. This suggests that the lake surface facilitates the persistence of different host-GV pairs from various ecological niches with different types of metabolisms.

In the monimolimnion, local GVMAGs were characterized by genes involved in the metabolisms of pyruvate, monosaccharides, and disaccharides. Additionally, the inverse correlation observed between the fatty acid degradation pathway in the monimolimnion and the synthesis and elongation of fatty acids in the mixolimnion implies a potential settling of organic matter from the lake's surface to the depths, made available for other microorganisms, including possible heterotrophic hosts [48, 57]. The monimolimnion and the GVMAGs likely adapted to this environment displayed higher abundance and greater correlations with genes involved in the pentose-glucuronate interconversion pathway. This pathway plays a role in various biosynthetic processes and notably in gluconeogenesis, a pathway in the biosynthesis of glucose from non-carbohydrate carbon substrates such as pyruvate during starvation [58, 59]. Together, these pathways imply a potential connection between the genetic makeup of GVMAGs and the metabolism of their potential hosts within the different habitats of the lake.

Finally, we detected VirRs from groups 1 and 2 along the entire water column of the lake. However, GVMAGs observed in the monimolimnion exhibited an increased VirR rate in their genetic makeup (74.2% compared to 50% in GVMAGs from alternative habitats). Notably, a significant number of them encoded both group 1 and 2 VirRs.

Contrary to ocean metagenomes, where group 1 VirRs are predominant [10], group 2 was most abundant in Lake A. A recently identified member of VirRs group 2 was suggested to have a potential role in modulating ion homeostasis within infected cells [12]. Another plausible scenario is that VirRs may impact ion currents across the membrane of infected protist hosts in response to light, potentially influencing their behavior, such as phototaxis [60, 61]. Finally, microbial rhodopsin have been observed in microbes ranging from archaea to eukaryotic microorganisms, including all major algal groups, likely enabling these organisms to supplement their energetic needs and/or guiding them toward better nutrient conditions to support growth in stressful conditions.

In the water column of Lake A, the DCM was characterized by extreme low light with PAR at and below detection. Still, the presence of photosynthetic organisms has already been detected at these anoxic depths in the lake and notably, the chlorosomes in the green sulfur bacteria have been shown to be extremely efficient at the blue end of the PAR spectrum [22]. The coding potential of GVMAGs for VirRs at such depth might indicate their potential to provide host species with additional light-gated ion channeling capabilities. Still, much remains to be understood regarding the ecological role of rhodopsins in viral infections in Lake A and in general.

## Conclusions

Remote, isolated, and permanently stratified Lake A is characterized by extreme physico-chemical gradients, making it an ideal environment to study the metabolic potential of *Nucleocytoviricota* within its stratified habitats. Each habitat hosts unique communities of GVs and potential protists hosts, with the genetic makeup of the GVs providing flexibility to adapt to local environmental conditions. The observed parallels between expected host metabolism and the genetic content of associated GVs suggest co-evolution and metabolic adaptation to the different habitats down the Lake A water column. These findings underscore the large viral diversity in polar lakes and the potential adaptability of GVs to local environmental conditions in such ecosystems. This diversity implies the key ecological importance of GVs in shaping local evolutionary history and biogeochemical cycles. The rapid warming of the Arctic is strongly impacting the limnology of far northern lakes and threatens to weaken the remarkable chemical stratification of ecosystems such as Lake A, which could lead to a loss of GV and host biodiversity and changes in biogeochemical functions.

## Supplementary Material

Supplementary_FIG_1_ismeco_ycae155

Supplementary_FIG_2_ismeco_ycae155

Supplementary_FIG_3_ismeco_ycae155

Extended_Data_Table_1_ycae155

Supplementary_figure_titles_and_captions_ismeco_ycae155

## Data Availability

The GVMAGs features and statistics used in this article are listed in Extended Data Table 1. Sequenced reads are available under Bioproject PRJNA1074374 (Canadian high-arctic lakes metagenomes) and GVMAGs sequences are available in figshare DOI: 10.6084/m9.figshare.25000571.

## References

[1] Mihara T , Koyano H, Hingamp P et al. Taxon richness of “*Megaviridae*” exceeds those of bacteria and archaea in the ocean. *Microb Environ* 2018;33:162–71. 10.1264/jsme2.ME17203 PMC6031395 29806626

[2] Wilson WH , Tarran GA, Schroeder D et al. Isolation of viruses responsible for the demise of an *Emiliania huxleyi* bloom in the English Channel. *J MarBiol Assoc UK* 2002;82:369–77. 10.1017/S002531540200560X

[3] Yau S , Seth-Pasricha M. Viruses of polar aquatic environments. *Viruses* 2019;11:189. 10.3390/v11020189 30813316 PMC6410135

[4] Koonin EV . Viruses and mobile elements as drivers of evolutionary transitions. *Phil Trans Royal Soc B: Biol Sci* 2016;371:20150442. 10.1098/rstb.2015.0442 PMC4958936 27431520

[5] Fischer MG , Allen MJ, Wilson WH et al. Giant virus with a remarkable complement of genes infects marine zooplankton. *Proc Natl Acad Sci USA* 2010;107:19508–13. 10.1073/pnas.1007615107 20974979 PMC2984142

[6] Monier A , Pagarete A, De Vargas C et al. Horizontal gene transfer of an entire metabolic pathway between a eukaryotic alga and its DNA virus. *Genome Res* 2009;19:1441–9. 10.1101/gr.091686.109 19451591 PMC2720186

[7] Schulz F , Yutin N, Ivanova NN et al. Giant viruses with an expanded complement of translation system components. *Science* 2017;85:82–5. 10.1126/science.aal4657 28386012

[8] Schulz F , Abergel C, Woyke T. Giant virus biology and diversity in the era of genome-resolved metagenomics. *Nat Rev Microbiol* 2022;20:721–36. 10.1038/s41579-022-00754-5 35902763

[9] Moniruzzaman M , Martinez-Gutierrez CA, Weinheimer AR et al. Dynamic genome evolution and complex virocell metabolism of globally-distributed giant viruses. *Nat Commun* 2020;11:1710. 10.1038/s41467-020-15507-2 32249765 PMC7136201

[10] Needham DM , Yoshizawa S, Hosaka T et al. A distinct lineage of giant viruses brings a rhodopsin photosystem to unicellular marine predators. *Proc Natl Acad Sci USA* 2019;116:20574–83. 10.1073/pnas.1907517116 31548428 PMC6789865

[11] Zabelskii D , Alekseev A, Kovalev K et al. Viral rhodopsins 1 are an unique family of light-gated cation channels. *Nat Commun* 2020;11. 10.1038/s41467-020-19457-7 33177509 PMC7659345

[12] Bratanov D , Kovalev K, Machtens JP et al. Unique structure and function of viral rhodopsins. *Nat Commun* 2019;10:4939. 10.1038/s41467-019-12718-0 31666521 PMC6821725

[13] Gallot-Lavallée L , Archibald JM. Evolutionary biology: viral rhodopsins illuminate algal evolution. *Curr Biol* 2020;30:1469–71. 10.1016/j.cub.2020.10.080 33352125

[14] Rozenberg A , Oppermann J, Wietek J et al. Lateral gene transfer of anion-conducting channelrhodopsins between green algae and giant viruses. *Curr Biol* 2020;30:4910–20. 10.1016/j.cub.2020.09.056 33065010

[15] Benites LF , Stephens TG, Van Etten J et al. Hot springs viruses at Yellowstone National Park have ancient origins and are adapted to thermophilic hosts. *Commun Biol* 2024;7:312. 10.1038/s42003-024-05931-1 38594478 PMC11003980

[16] Schulz F , Roux S, Paez-Espino D et al. Giant virus diversity and host interactions through global metagenomics. *Nature* 2020;578:432–6. 10.1038/s41586-020-1957-x 31968354 PMC7162819

[17] Meng L , Delmont TO, Gaïa M et al. Genomic adaptation of giant viruses in polar oceans. *Nat Commun* 2023;14:6233. 10.1038/s41467-023-41910-6 37828003 PMC10570341

[18] Pitot TM , Rapp JZ, Schulz F et al. Distinct and rich assemblages of giant viruses in Arctic and Antarctic lakes. *ISME Commun* 2024;4:ycae048. 10.1093/ismeco/ycae048 38800130 PMC11128243

[19] Vincent WF , Laybourn-Parry J (eds). Polar Lakes and Rivers: Limnology of Arctic and Antarctic Aquatic Ecosystems. Oxford University Press, Oxford, 2008. Online edn. 10.1093/acprof:oso/9780199213887.001.0001

[20] Vincent WF , Fortier D, Levesque E et al. Extreme ecosystems and geosystems in the Canadian high Arctic: Ward Hunt Island and vicinity. *Ecoscience* 2011;18:236–61. 10.2980/18-3-3448

[21] Zhang L , Meng L, Fang Y et al. Spatiotemporal dynamics of giant viruses within a deep freshwater lake reveal a distinct dark-water community. *ISME J* 2024;18:wrae182. 10.1093/ismejo/wrae182 39312489 PMC11465185

[22] Vigneron A , Cruaud P, Culley AI et al. Genomic evidence for sulfur intermediates as new biogeochemical hubs in a model aquatic microbial ecosystem. *Microbiome* 2021;9:46. 10.1186/s40168-021-00999-x 33593438 PMC7887784

[23] Charvet S , Vincent W, Comeau A et al. Pyrosequencing analysis of the protist communities in a high Arctic meromictic lake: DNA preservation and change. *Front Microbiol* 2012;3:422. 10.3389/fmicb.2012.00422 23267353 PMC3526917

[24] Comeau AM , Harding T, Galand PE et al. Vertical distribution of microbial communities in a perennially stratified Arctic lake with saline, anoxic bottom waters. *Sci Rep* 2012;2:604. 10.1038/srep00604 22930670 PMC3428602

[25] Labbé M , Girard C, Vincent WF et al. Extreme viral partitioning in a marine-derived high Arctic lake. *mSphere* 2020;5:e00334–20. 10.1128/msphere.00334-20 32404515 PMC7227771

[26] Gibson JAE , Vincent WF, Van Hove P et al. Geochemistry of ice-covered, meromictic Lake a in the Canadian high Arctic. *Aquat Geochem* 2002;8:97–119. 10.1023/A:1021317010302

[27] Cruaud P , Vigneron A, Fradette MS et al. Open the SterivexTM casing: an easy and effective way to improve DNA extraction yields. *Limnol Oceanogr Methods* 2017;15:1015–20. 10.1002/lom3.10221

[28] Mueller JA , Culley AI, Steward GF. Variables influencing extraction of nucleic acids from microbial plankton (viruses, bacteria, and protists) collected on nanoporous aluminum oxide filters. *Appl Environ Microbiol* 2014;80:3930–42. 10.1128/AEM.00245-14 24747903 PMC4054216

[29] Rohart F , Gautier B, Singh A et al. mixOmics: an R package for ‘omics feature selection and multiple data integration. *PLoS Comput Biol* 2017;13:e1005752. 10.1371/journal.pcbi.1005752 29099853 PMC5687754

[30] Kang DD , Li F, Kirton E et al. MetaBAT 2: an adaptive binning algorithm for robust and efficient genome reconstruction from metagenome assemblies. *PeerJ* 2019;7:e7359. 10.7717/peerj.7359 31388474 PMC6662567

[31] Olm MR , Brown CT, Brooks B et al. DRep: a tool for fast and accurate genomic comparisons that enables improved genome recovery from metagenomes through de-replication. *ISME J* 2017;11:2864–8. 10.1038/ismej.2017.126 28742071 PMC5702732

[32] Pitot TM , Brůna T, Schulz F. Conservative taxonomy and quality assessment of giant virus genomes with GVClass. *NPJ Viruses* 2024;2:60. 10.1038/s44298-024-00069-7 PMC11721457 40295812

[33] Ha AD , Aylward FO. Automated classification of giant virus genomes using a random forest model built on trademark protein families. *NPJ Viruses* 2024;2:9. 10.1038/s44298-024-00021-9 PMC11721082 40295679

[34] Aylward FO , Moniruzzaman M. Viralrecall—a flexible command-line tool for the detection of giant virus signatures in ‘omic data. *Viruses* 2021;13:150. 10.3390/v13020150 33498458 PMC7909515

[35] Conway JR , Lex A, Gehlenborg N. UpSetR: an R package for the visualization of intersecting sets and their properties. *Bioinformatics* 2017;33:2938–40. 10.1093/bioinformatics/btx364 28645171 PMC5870712

[36] Wickham H . ggplot2. *Wiley Interdiscip Rev Comput Stat* 2011;3:180–5. 10.1002/wics.147

[37] Bushnell B , Rood J, Singer E. BBMerge—accurate paired shotgun read merging via overlap. *PLoS One* 2017;12:e0185056. 10.1371/journal.pone.0185056 29073143 PMC5657622

[38] Yilmaz P , Parfrey LW, Yarza P et al. The SILVA and ‘all-species living tree project (LTP)’ taxonomic frameworks. *Nucleic Acids Res* 2014;42:643–8. 10.1093/nar/gkt1209 24293649 PMC3965112

[39] Besemer J , Lomsadze A, Borodovsky M. GeneMarkS: a self-training method for prediction of gene starts in microbial genomes. Implications for finding sequence motifs in regulatory regions. *Nucleic Acids Res* 2001;29:2607–18. 10.1093/nar/29.12.2607 11410670 PMC55746

[40] Cantalapiedra CP , Hernández-Plaza A, Letunic I et al. eggNOG-mapper v2: functional annotation, orthology assignments, and domain prediction at the metagenomic scale. *Mol Biol Evol* 2021;38:5825–9. 10.1093/molbev/msab293 34597405 PMC8662613

[41] Hamilton NE , Ferry M. Ggtern: ternary diagrams using ggplot2. *J Stat Softw* 2018;87:1–17. 10.18637/jss.v087.c03

[42] Camargo AP , Roux S, Schulz F et al. Identification of mobile genetic elements with geNomad. *Nat Biotechnol* 2024;42:1303–12. 10.1038/s41587-023-01953-y 37735266 PMC11324519

[43] Tennekes M , Ellis P. Treemap: Treemap visualization. *R package version* 2017;2:2–4.

[44] Mistry J , Chuguransky S, Williams L et al. Pfam: the protein families database in 2021. *Nucleic Acids Res* 2021;49:412–9. 10.1093/nar/gkaa913 PMC7779014 33125078

[45] Ha AD , Moniruzzaman M, Aylward FO. High transcriptional activity and diverse functional repertoires of hundreds of giant viruses in a coastal marine system. *mSystems* 2021;6:1128. 10.1128/msystems.00293-21 PMC8407384 34254826

[46] Lemoine F, Gascuel O . Gotree/Goalign: toolkit and Go API to facilitate the development of phylogenetic workflows. *NAR Genomics and Bioinformatics* 2021;3:lqab075. 10.1093/nargab/lqab075 PMC8356961 34396097

[47] Letunic I , Bork P. Interactive tree of life (iTOL) v5: an online tool for phylogenetic tree display and annotation. *Nucleic Acids Res* 2021;49:293–6. 10.1093/nar/gkab301 PMC8265157 33885785

[48] Antoniades D , Veillette J, Martineau M-J et al. Bacterial dominance of phototrophic communities in a high Arctic lake and its implications for paleoclimate analysis. *Polar Sci* 2009;3:147–61. 10.1016/j.polar.2009.05.002

[49] Aylward FO , Abrahão JS, Brussaard CPD et al. Taxonomic update for giant viruses in the order Imitervirales (phylum *Nucleocytoviricota*). *Arch Virol* 2023;168:283. 10.1007/s00705-023-05906-3 37904060 PMC11230039

[50] Irwin NAT , Pittis AA, Richards TA et al. Systematic evaluation of horizontal gene transfer between eukaryotes and viruses. *Nat Microbiol* 2022;7:327–36. 10.1038/s41564-021-01026-3 34972821

[51] Moniruzzaman M , Weinheimer AR, Martinez-Gutierrez CA et al. Widespread endogenization of giant viruses shapes genomes of green algae. *Nature* 2020;588:141–50. 10.1038/s41586-020-2924-2 33208937

[52] Raoult D , Audic S, Robert C et al. The 1.2-megabase genome sequence of Mimivirus. *Science* 2004;306:1344–50. 10.1126/science.1101485 15486256

[53] Comeau AM , Vincent WF, Bernier L et al. Novel chytrid lineages dominate fungal sequences in diverse marine and freshwater habitats. *Sci Rep* 2016;6:30120. 10.1038/srep30120 27444055 PMC4957111

[54] Cao M , Bi G, Mao Y et al. The first plastid genome of a filamentous taxon ‘*Bangia*’ sp. OUCPT-01 in the Bangiales. *Sci Rep* 2018;8:10688. 10.1038/s41598-018-29083-5 30013114 PMC6048033

[55] Bégin PN , Tanabe Y, Kumagai M et al. Extreme warming and regime shift toward amplified variability in a far northern lake. *Limnol Oceanogr* 2021;66:17–29. 10.1002/lno.11546

[56] Xu F , Wu X, Jiang LH et al. An organelle K++ channel is required for osmoregulation in *Chlamydomonas reinhardtii*. *J Cell Sci* 2016;129:3008–14. 10.1242/jcs.188441 27311484

[57] Tomkins JD , Lamoureux SF, Antoniades D et al. Sedimentology of perennial ice-covered, meromictic Lake A, Ellesmere Island, at the northern extreme of Canada. *Can J Earth Sci* 2009;46:83–100. 10.1139/E09-008

[58] Hanson RW , Owen OE. Gluconeogenesis. In: Lennarz W.J., Lane M.D. (eds.), Encyclopedia of Biological Chemistry, Second edn. Waltham: Academic Press, 2013, 381–6.

[59] Nelson DL , Cox MM. Lehninger Principles of Biochemistry. New York: Worth Publishers, 2000.

[60] Philosof A , Béjà O. Bacterial, archaeal and viral-like rhodopsins from the Red Sea. *Environ Microbiol Rep* 2013;5:475–82. 10.1111/1758-2229.12037 23754728

[61] Yutin N , Koonin EV. Proteorhodopsin genes in giant viruses. *Biol Direct* 2012;7:34. 10.1186/1745-6150-7-34 23036091 PMC3500653

