# Supplementary figures and images for: Viral niche-partitioning: comparative genomics of giant viruses across environmental gradients in a high Arctic freshwater-saltwater lake
Source: ISME Commun. 2024 Dec 8;5(1):ycae155. doi: 10.1093/ismeco/ycae155 (PMC11745019; doi:10.1093/ismeco/ycae155)

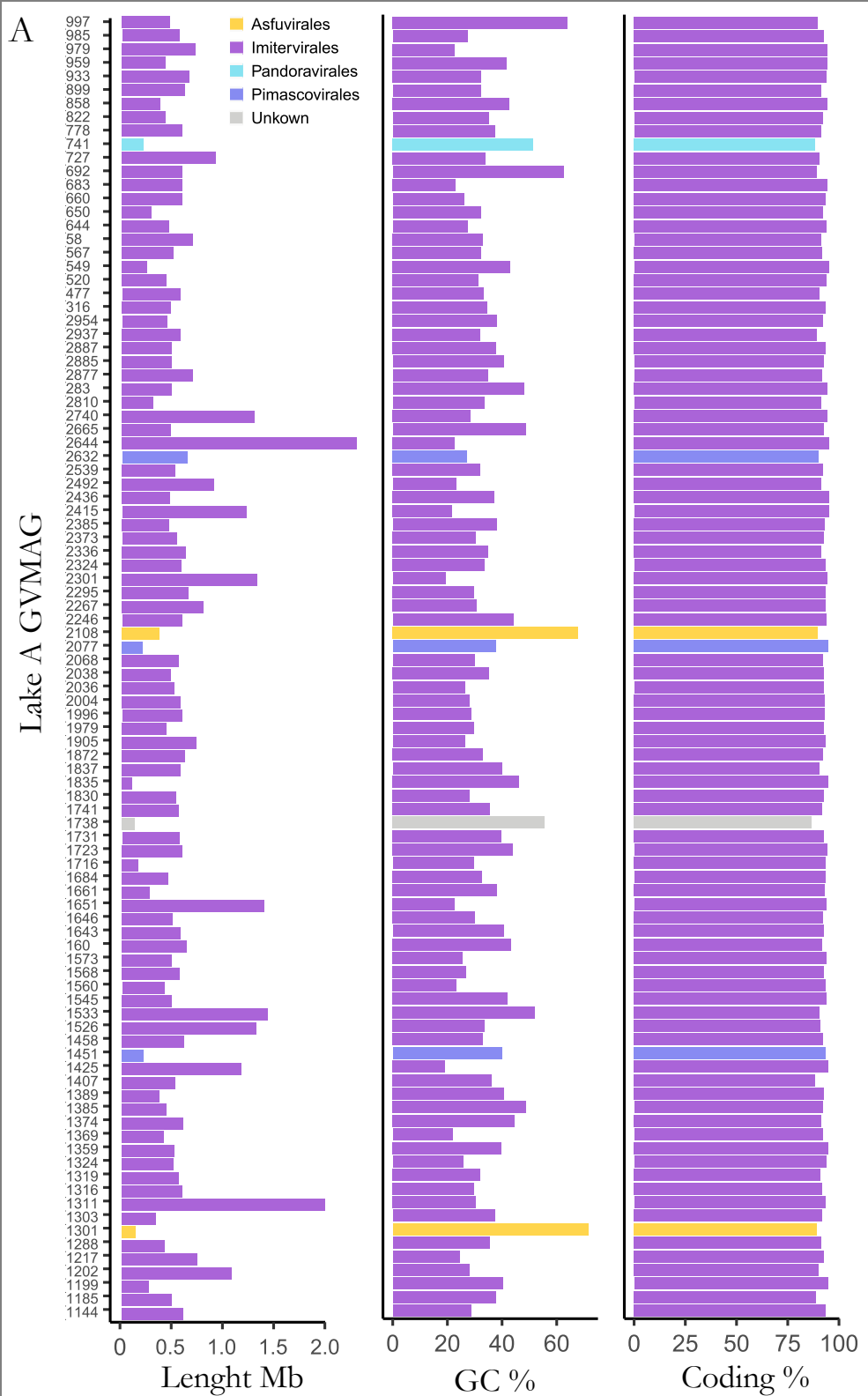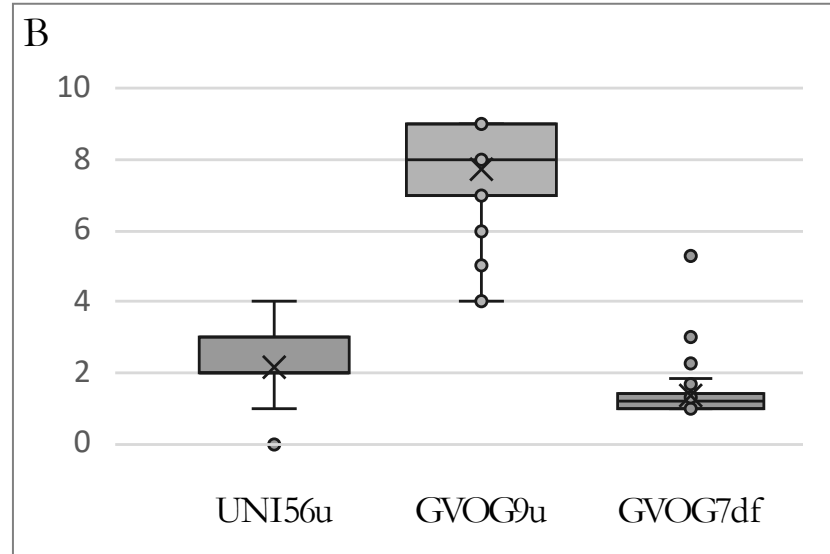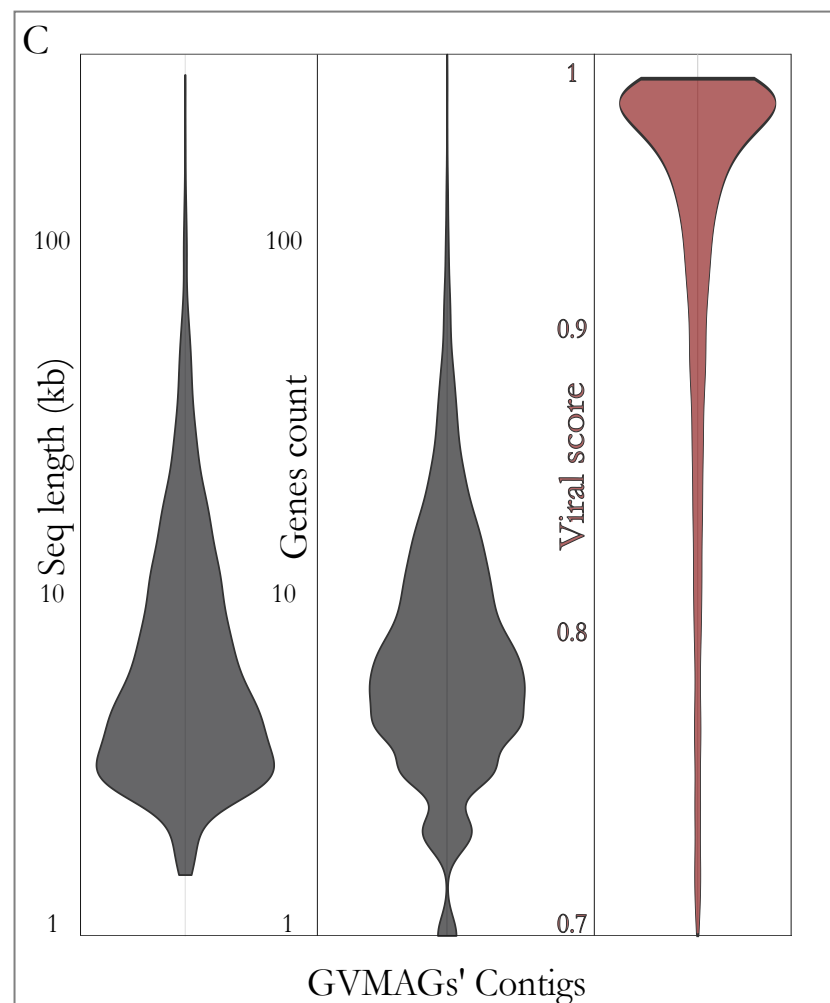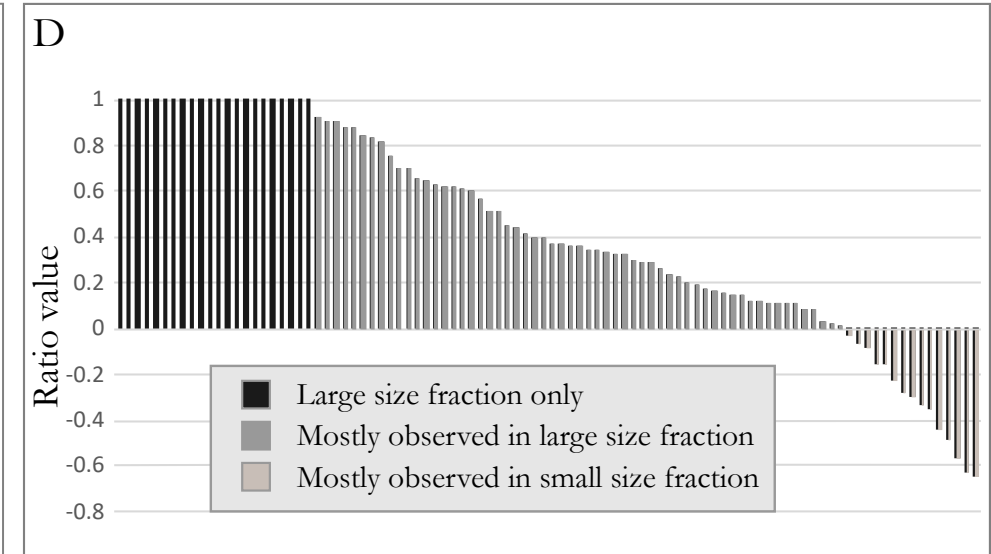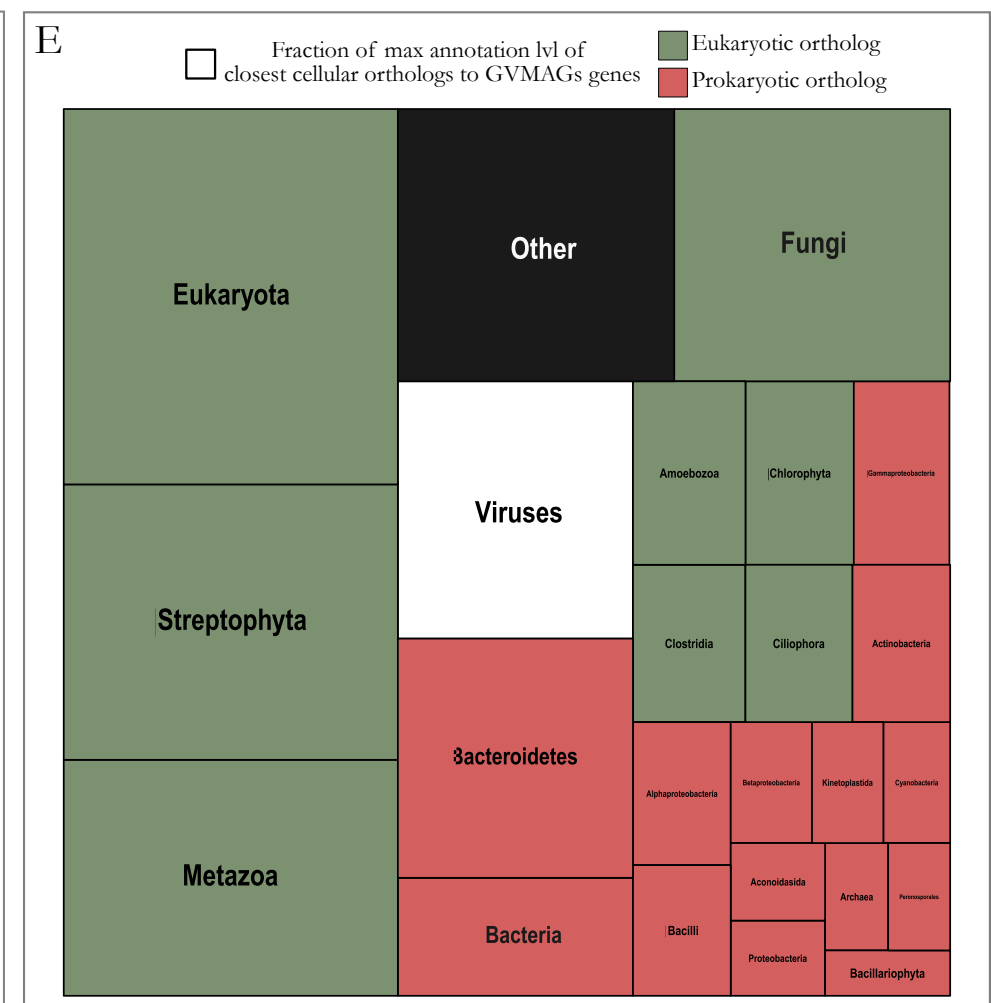

Supplement: Supplementary_FIG_1_ismeco_ycae155 [file supplementary_fig_1_ismeco_ycae155.pdf]

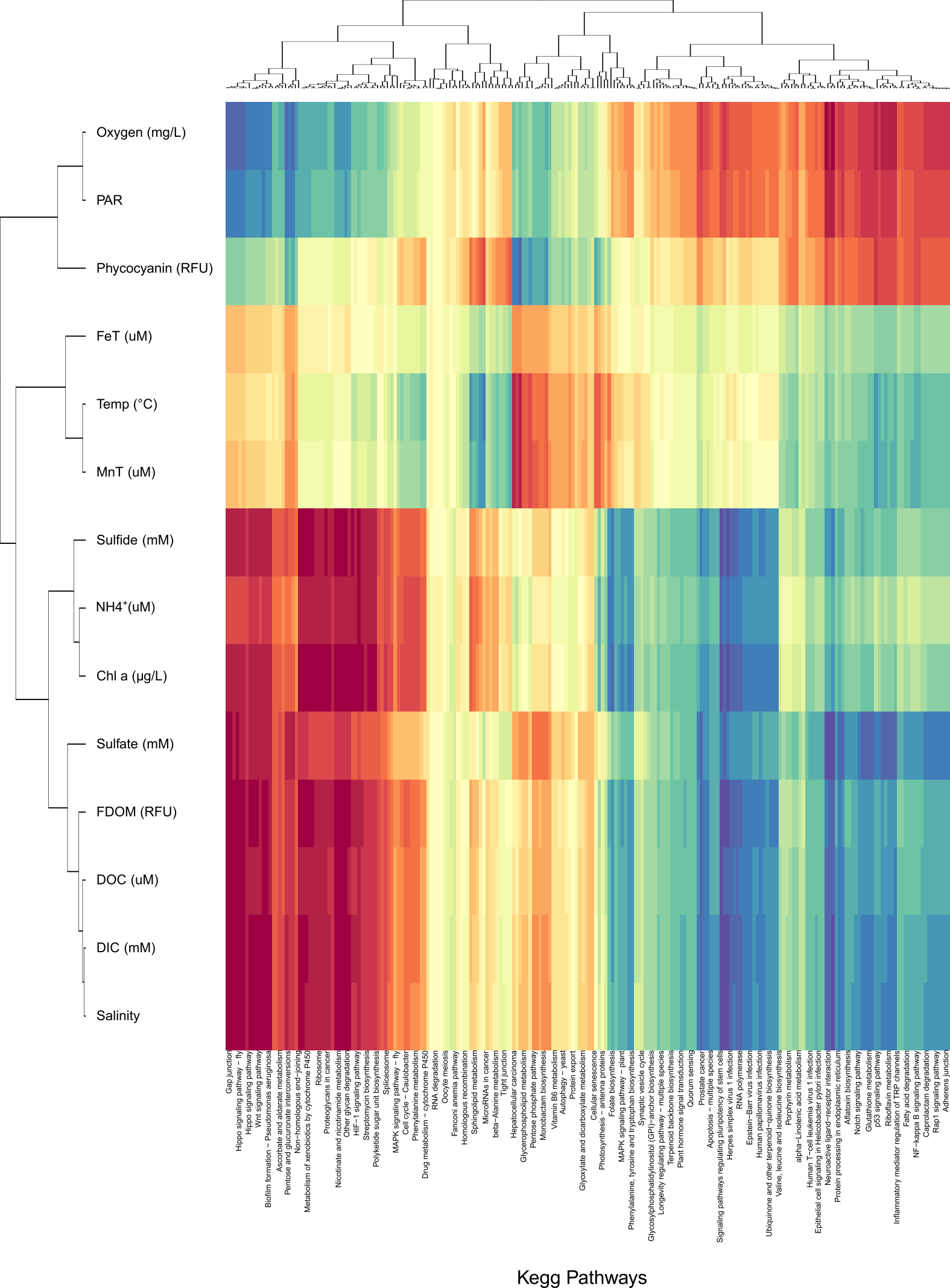

Supplement: Supplementary_FIG_2_ismeco_ycae155 [file supplementary_fig_2_ismeco_ycae155.pdf]

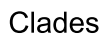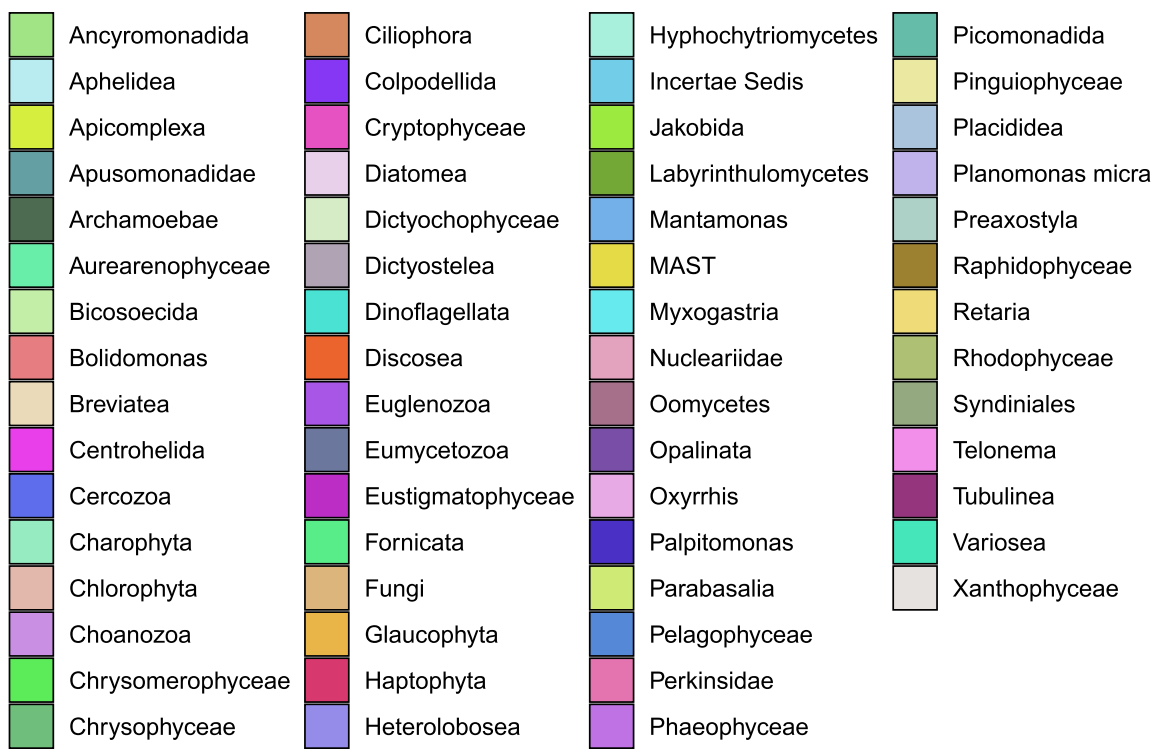

Supplement: Supplementary_FIG_3_ismeco_ycae155 [file supplementary_fig_3_ismeco_ycae155.pdf]
