# Supplementary material for: Viral niche-partitioning: comparative genomics of giant viruses across environmental gradients in a high Arctic freshwater-saltwater lake
Source: ISME Commun. 2024 Dec 8;5(1):ycae155. doi: 10.1093/ismeco/ycae155 (PMC11745019; doi:10.1093/ismeco/ycae155)
Supplement: Supplementary_figure_titles_and_captions_ismeco_ycae155 [file supplementary_figure_titles_and_captions_ismeco_ycae155.docx]

*Supplementary Fig. 1: GVMAGs features****.*** **A** Features of GVMAGs (taxonomy, length in Mb, GC content, and coding percentage). For accession numbers of GVMAGs, see supplementary Table 1. **B** Quality markers of the MAGs (universal cellular housekeeping genes, set of 9 giant virus orthologous groups, duplication factor of GVOG7). **C** Features of contigs identified as highly probable viral by geNomad (length in kb, gene count, viral score). **D** Distribution ratio of MAGs between the large size fraction (>0.22 μm) and the small size fraction (<0.22 μm). **E** Overall gene taxonomy of total predicted GVMAG proteins.

*Supplementary Fig. 2: GeNomad viral contigs and metadata correlations.* Clustered Image Map (CIM) heatmap displays Pearson correlation coefficients, revealing the relationship between metadata and the abundance of KEGG pathways. This analysis specifically examined contigs predicted as viral by geNomad across the water column, rather than considering the entire GVMAGs. NH4^+^: ammonium-N.

*Supplementary Fig. 3: Spatial dynamics of eukaryotic cells in the lake A.* Bar chart of the relative abundance of eukaryotic clades along Lake A water column.
